# An Essay on a New Method of Adapting Artificial Teeth

**Published:** 1854-04

**Authors:** 


### ARTICLE XII.

*An Essay on a New Method of Adapting Artificial Teeth.* By Dr. A. Fitzpatrick, Surgeon Dentist, &c., &c. Thacker & Co., London, pp. 40.

The author of this essay resided many years in India, where, if the original correspondence can be relied upon, he practiced his profession with credit and success. The doctor, in his introduction to his essay, starts at full speed, publishes his discovery, and at once and for ever claims originality, in the construction of plates for artificial work. This, however, should be excused, considering the author was somewhat isolated in his position, and may be said not quite "up" to the various improvements introduced by his American and European brethren. The introduction says: "My object in publishing this little treatise on *my* recently discovered mode of adapting artificial teeth to the human mouth, is that, being convinced of the importance of my discovery, I should lose no time in bringing it at once and prominently to the knowledge of the profession to which I belong, and of the public at large, for whose benefit it has been deeply and carefully studied, and is now openly and unreservedly published and divulged. I confess that at first I thought of security to myself by a patent, the advantages and profits of the invention, and so monopolizing the harvest which I believe is to be reaped from my successful labors and discovery; but I soon abandoned this idea, reflecting that the first duty of an educated and enlightened professional man is, to make known to the world, for the benefit of mankind, any improvement in art or science that can relieve the sufferings of his fellow creatures, or conduce to the alleviation of their afflictions, without reference to his own selfish interests or personal aggrandizement." A Harvey, a Franklin, or a Jenner, could not have spoken more to the purpose, or have attached a greater amount of importance to their LITTLE discoveries, than the present writer of this essay. If the doctor means in his observations respecting patents, that he is opposed to specifics for alleviating or curing disease, suspending the nervous functions during surgical operations, and such like, we most willingly agree with him.

But how comes it that the doctor did think of a patent, and seeking "a golden harvest," he seems to blow hot and cold with the same breath, for he says, "the advantages of improvements in the strictly mechanical arts, and the profits of invention, ingenuity in handicraft trades or occupations, ought, perhaps, to be secured to the discoverer for such a period of time as will reward his labor, and so serve as an inducement to others to prosecute useful inquiries, and to direct their energies toward *honorable* and *lucrative* discoveries; but I take a wide view of the duty which he owes to society, whose profession it is to alleviate physical sufferings, remove deformity, or check disease."

Then why, we ask, did the author, if he sincerely entertain these views, think at first of patenting his invention, he cannot be aware that true dental mechanics strictly belong to the mechanical sciences, and as such, its position has been assigned by common consent by all writers upon this branch of dentistry.

We would infinitely prefer that a practitioner who has made an important discovery in mechanical dentistry, should avail himself of the patent laws, to his *secretly* confining the use of his invention to his own individual practice. Moreover, the doctor must be aware there are many trifles which, in the estimation of the party *interested should be patented*, but when brought into actual practice, and its value estimated by other parties, have been found wanting in the scale of utility, and consequently worthless; whilst others again are mere insignificant *specs* of science, that it would be perfectly absurd to think of a patent.

The author proceeds. "All the advantage I desire from my discovery, viz. the credit of priority in having introduced a total change into that branch of the dental art with which I am about to occupy the reader, and of having revolutionized the entire mechanical system of one of the most important operations of the dentist, that of fitting artificial teeth into the mouth with credit and satisfaction to himself, and relief and comfort to his patients." This kind of egotistical philosophy reads well in print, and probably sounds well, if well expressed, to patients in the operating room. We much question, however, whether the more sober thinking portion of dental practitioners will attach that amount of importance to the discovery as the doctor has by "having revolutionized the whole mechanical system."

Unquestionably, credit is due to the author, in treating this subject scientifically, and in placing it prominently before the profession, but we are bound, as journalists, to state that the whole merit of introducing this kind of work does not entirely belong to Dr. Fitzpatrick. We remember having seen plates cut in a somewhat similar manner, and in use some eight or ten years since, having been manufactured by a Mr. Lukyn. More recently, in conversation with a brother professional, (Mr. Levison,) this gentleman also stated he had one by him made by the same party, and which had been worn by his patient for some years. As we before observed, we accord to the author the merit of having scientifically treated the subject, but whether its adoption in practice will warrant that amount of importance, in a scientific point of view, as attached by the author, remains to be seen.

Dr. Fitzpatrick, from his own account, has undergone intense mental and physical exertions in bringing this discovery to a successful issue, for he says, "with all the application and reflection that I could devote to what was to me a very interesting subject, it was a considerable time before I could arrive at even the fundamental principles of my discovery, and much more time, even when the frame work of my theory was arranged, was consumed in filling up mechanical deficiencies, or evading the impediments momentarily cast in my way by anatomical or physiological difficulties." The author appears to have the most extraordinary ideas of his professional brethren, when he pictures to himself the *opposition* his new plan will meet with, owing to "the old wedded opinions and theories," and the increase of labor and expense necessary in making the plates. He may make himself perfectly happy upon that point, if this new system is found to possess only one-half the merit the learned doctor claims for it; he may rest assured the profession, either in Europe or America, will not oppose its adoption from either an increase of expense or labor, he observes: "In making public my new mode of fixing artificial teeth in the mouth, I am aware that I shall meet with opposition from many who are wedded to old opinions and theories, and to the routine of the practice hitherto in use in the profession to which I belong. Prejudice with some, and increase of labor and expense with others, in some cases both combined, will militate against the sudden and successful introduction of the great and important innovation which I desire to introduce into the mechanical branch of our profession."

After quoting several authors relative to the value and importance of artificial teeth as auxiliaries to health, he makes the following sensible observation upon the sense of taste: "Now, as it is evident that the delicate discrimination in the taste of different articles of food may be so materially effected by the natural state of the palate, how much more must it necessarily be influenced when art interferes with nature, by introducing into the mouth a foreign substance, either metallic or otherwise, of such a form as to completely cover the palate, where nature has, in her all-wise beneficence, taken care to provide nerves of exquisite sense, which ramify over it from their main trunks, arising from behind their central incisors, and others distributed laterally, proceeding from the posterior portion of the palate, besides which, in order that the food may have as extended a plain of action as possible, the surface of the plate is corrugated, thus considerably enlarging the expanse of membrane exposed to the contact of the different aliments."

The author describes the various kinds of plates now employed for fixing artificial teeth upon, and concludes his essay by claiming the following advantages for his own plan:

"The first advantage that I obtain by this plate is, that the palate being uncovered, can maintain its healthy state, which it cannot maintain if perpetually covered."

"The second advantage, that the teeth and plate do not require to be taken out at night, and by this the expense of night teeth, so often necessary for the comfort of the patient under the present system, may be avoided."

"The third advantage is, that the palate not being covered with metal plate, no food can get between it and such plate, and consequently no irritation of the mucous membrane can take place."

"The fourth advantage is, that the mechanical friction, or the galvanic influence that might take place, is perfectly avoided by giving free action to the circulation in the palate."

"The fifth advantage is, that whereas, under the present system, in cases in which gold could not be employed, in consequence of its hard and unyielding nature, patients were obliged to have recourse to bone; but, on the other hand, as my plates give every freedom to the palate, the use of bone can be entirely done away with."

"The sixth advantage is, that the parts supplied by the principal nerves being uncovered, and the palate exposed to the influence of the saliva and tongue, the faculty of taste is not impaired. This will not be considered of trifling importance to those who are fond of the pleasures of the table."

"The seventh, and not the least advantage, is, that the front of the palate being uncovered, there is no impediment to the speech, as occurs in many cases where the palate is covered by the plates now in use."

"The eighth advantage that they possess is, that they do not require to be taken out daily, as no food can lodge between them and the plate, it being necessarily carried off by the tongue."

"The ninth advantage is, that though strong, they are light, and have quite as much strength as if the palate were covered."

"The tenth advantage is, that they give less trouble to the patient on their being first placed in the mouth, and consequently the tongue being perfectly free, and finding no obstacle, gets used to them at once."

The author's remarks upon the employment of cheap materials, inferior workmanship, &c., in the manufacture of mechanical work are excellent, he is evidently only partially acquainted with this subject, for we can assure him, there are practitioners of a higher grade in public estimation than the low, cunning, advertising cheap fraternity, who do not scruple to insert pieces of work in the mouth of the most inferior workmanship and worse fits, yet charging the most exorbitant prices. As for their fillings, its the *exception* with two or three of these worthies, when they do *attempt* to fill lateral cavities with gold, most generally the *amalgams* is their glorious compound. Yet some one of these men calls himself the "Dental Mæcenus" of the "Sabine Farm." Alas—poor Horace, to what a wretched state of degradation thou hast fallen.

Having now perused Dr. Fitzpatrick's essay on his adaptation of perforated plates, we must again repeat, the principle is not entirely original, the author unquestionably deserves much credit in bringing the subject prominently before the profession and treating it upon scientific principles, and we have no doubt that our brother professionals will soon test the value of the invention, and make their report accordingly. R.

### SELECTED ARTICLES.

